# Research Progress on the Anti-Cancer Effects of *Astragalus membranaceus* Saponins and Their Mechanisms of Action

**DOI:** 10.3390/molecules29143388

**Published:** 2024-07-18

**Authors:** Feiya Sheng, Siyu Yang, Mi Li, Jiaojiao Wang, Lianghong Liu, Lele Zhang

**Affiliations:** 1 College of Pharmacy, Chengdu University, Chengdu 610106, China; shengfeiya@cdu.edu.cn (F.S.); 19511878892@163.com (S.Y.); limimmmmm@126.com (M.L.); 18708568296@163.com (J.W.); 2 School of Pharmaceutical Sciences, Hunan University of Medicine, Huaihua 418000, China; 3 School of Basic Medical Sciences, Chengdu University, Chengdu 610106, China

**Keywords:** *Astragalus membranaceus* saponins, antitumor, proliferation, apoptosis, metastasis, migration, tumor microenvironment, signaling pathway

## Abstract

*Astragalus membranaceus* saponins are the main components of *A. membranaceus*, a plant widely used in traditional Chinese medicine. Recently, research on the anti-cancer effects of *A. membranaceus* saponins has received increasing attention. Numerous in vitro and in vivo experimental data indicate that *A. membranaceus* saponins exhibit significant anti-cancer effects through multiple mechanisms, especially in inhibiting tumor cell proliferation, migration, invasion, and induction of apoptosis, etc. This review compiles relevant studies on the anti-cancer properties of *A. membranaceus* saponins from various databases over the past two decades. It introduces the mechanism of action of astragalosides, highlighting their therapeutic benefits in the management of cancer. Finally, the urgent problems in the research process are highlighted to promote *A. membranaceus* saponins as an effective drug against cancer.

## 1. Introduction

According to the World Health Organization, cancer is a group of illnesses that can impact any area of the body, characterized by rapidly proliferating, aberrant cells that infect neighboring body parts and spread to other organs [1]. By 2040, an estimated 28.4 million new cases of cancer are expected worldwide [1,2]. Particularly, the Human Development Index (HDI) of low- and middle-income nations is predicted to experience the most significant relative increases [3,4], posing a serious risk to public health. Currently, various cancer treatment modalities exist, including surgery, chemotherapy, targeted therapy [5,6], and immunotherapy [7]. However, these strategies have several limitations, including various adverse effects [8,9], medication resistance [10,11], and slow reaction times [12,13]. Therefore, there is an urgent need to explore new therapeutic drugs and mechanisms to accurately target tumors.

*Astragalus membranaceus*, a species of legumes mainly distributed in the Northern Hemisphere, South America, and Africa [14,15,16], is widely used in the treatment of many types of cancer. It is primarily produced in China, South Korea, Mongolia, and other regions [15,16]. *Astragalus membranaceus* exhibits multiple functions, including improving lung immunity, reducing the occurrence of thrombosis, lowering blood pressure, protecting the heart, dilating blood vessels, enhancing blood circulation, and regulating blood sugar [17,18]. Moreover, recent research has demonstrated the significant antitumor effects of *A. membranaceus* saponins against various cancer types, including gastric cancer (GC) [19], breast cancer [20], lung cancer, non-small cell lung cancer (NSCLC), liver cancer, hepatocellular carcinoma (HCC), colorectal cancer (CRC) [21], prostate cancer (PCA), vulvar squamous cell carcinoma (VSCC), and cervical cancer [22,23]. Notably, current research is ongoing to determine the mechanism underlying the regulatory actions of *A. membranaceus* saponin.

The present review aimed to summarize and synthesize research on the antitumor mechanism of *A. membranaceus* saponin from PubMed, Web of Science, and China National Knowledge Infrastructure (CNKI). This study also described the mechanisms that underlie the therapeutic benefits of *A. membranaceus* saponin in the management of tumors.

## 2. Triterpenoid Saponins

Saponins are the primary active components of *A. membranaceus*, predominantly comprising triterpenoids [24]. These saponins are classified into cyclobutane type and oleanane type, totaling 161 species [25,26]. Cyclobutane-type saponins feature substituents such as β-D-glucopyranosyl (Glc), β-D-xylopyranosyl (Xyl), α-L-rhamnopyranosyl (Xyl), α-L-xylopyranosyl (Xyl), glucopyranosyl (Glc), β-D-xylopyranosyl (Xyl), and α-L-rhamnopyranosyl (Rha) [25], often with acyclic side chains at the 17 position and oxygen-containing groups at the 24 position [27,28]. The hydroxyl group at the 20 and 24 positions can be easily dehydrated to form a furan ring, such as Astragaloside I~IV, VII, and cycloaraloside [29,30]. In oleanane-type saponins, the methyl at the 23 position is replaced by β-hydroxymethyl, and the representative compounds are soyasaponin and astragaloside-VIII (AS-VIII), amongst others [31,32]. When expressed in yeast, the *A. membranaceus AmOSC2* and *AmOSC3* genes can biosynthesize *A. membranaceus* saponins. This recent finding provides new information about the synthesis and possible use of saponins from *A. membranaceus* in the future [33].

**Figure 1:**
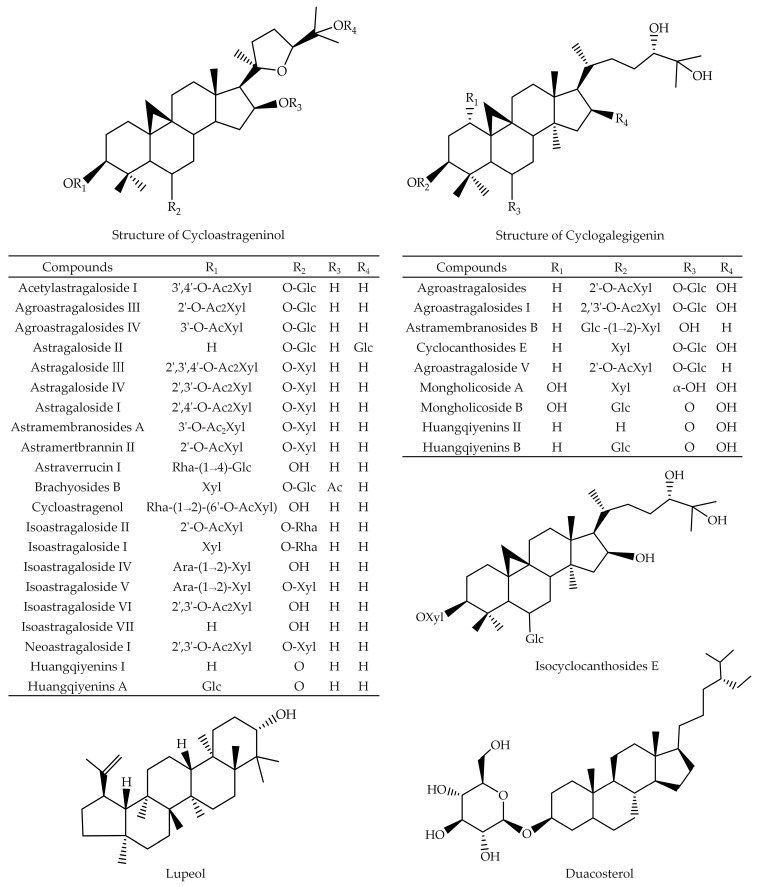
Chemical structures of the cycloastrageninol, cyclogalegigenin, isocyclocanthoside E, lupeol, and duacosterol isolated from *A. membranaceus*.

**Figure 2:**
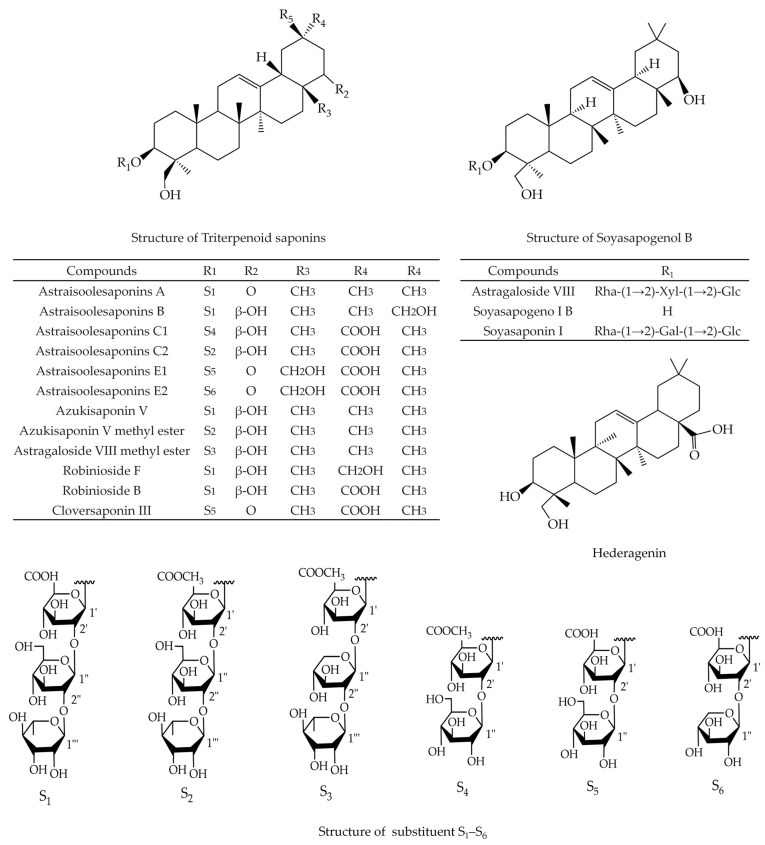
Chemical structures of the triterpenoid saponins, soyasapogenol B, and hederagenin isolated from *A. membranaceus*.

*A. membranaceus* saponins, including AS-II, AS-III, AS-IV, soyasaponin I (SsaI), cycloastragenol (CAG), lupeol, daucosterol (DS), and hederagenin (HDG), exhibit noteworthy inhibitory effects on various cancer cells, including uterus, lung, breast, colon, CRC, HCC, GC, PCA, and VSCC [15]. Chemical structures of the *A. membranaceus* saponins with anti-cancer activity are listed in Figure 1 and Figure 2.

## 3. Anti-Cancer Effect of the Extracts of *A. membranaceus* Saponins

**Figure 3:**
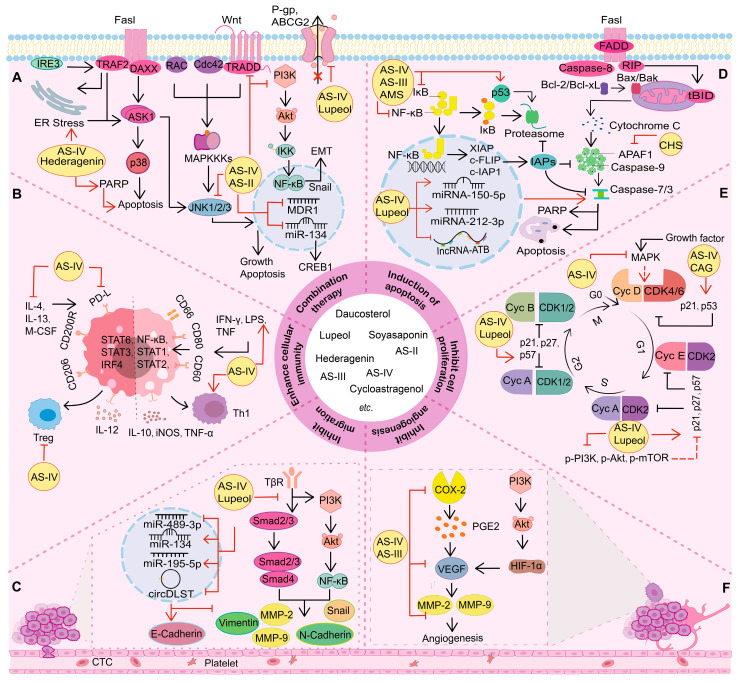
The mechanisms of action of the *A. membranaceus* saponins with anti-cancer activity. (**A**) *A. membranaceus* saponins synergize with other drugs to exert synergistic antitumor effects. *A. membranaceus* saponins increase cellular sensitivity to drugs by modulating the MAPK, PI3K/Akt signaling pathway, etc. (**B**) *A. membranaceus* saponins regulate immune function. *A. membranaceus* saponins regulate the expression of IL-6, IL-10, IFN-γ, and IL-12 and inhibit Treg cell activity, etc. (**C**) *A. membranaceus* saponins regulate signaling pathways such as TGF-β/Smad and suppress tumor cell migration and invasion. (**D**) Effects of *A. membranaceus* saponins on apoptosis. *A. membranaceus* saponins regulate the expression of Cyt C, Omi, Bax, and Caspase-3; activate the Fas/FasL pathway; and inhibit the expression of anti-apoptotic proteins such as XIAP, MCL1, and CFLIP. (**E**) Effects of *A. membranaceus* saponins on tumor cell proliferation. *A. membranaceus* saponins regulate CDK, Cyclin, p21, p53, etc., resulting in cell cycle arrest. (**F**) Effects of *A. membranaceus* saponins on angiogenesis. *A. membranaceus* saponins inhibit tumor angiogenesis by inhibiting VEGF, MMP-2, MMP-9, CD31, etc.

*A. membranaceus* saponins have been proven to exhibit anti-cancer potential in various cancer types. The mechanisms of action include inhibiting tumor cell proliferation, migration, and invasion; induction of apoptosis; regulation of autophagy; inhibition of angiogenesis; boosting immunity, etc. (Table 1, Figure 3).

### 3.1. Inhibition of Cell Proliferation

One crucial indicator of malignant tumors is the continuous proliferation of tumor cells [118]. By controlling the cell cycle and associated signaling pathways, AS-IV, lupeol, CAG, and DS can inhibit lung cancer, liver cancer, colon cancer, CRC, and VSCC.

Numerous proteins and genes are regulated during the cell cycle. These proteins consist of mitotic checkpoint proteins (M proteins), Cyclin-dependent kinase (CDK), Cyclin-dependent kinase inhibitors, and Cyclin [119]. Particularly, AS-IV inhibits the expression of Cyclin D1 and CDK4 in CRC cells, resulting in cell cycle arrest in the G0/G1 phase. Additionally, tumor suppressor genes can also block the cell cycle. Zhao et al. [39] demonstrated that the expression of the transforming growth factor-β-type II receptor *(TGF-βRII)* and *SMAD4* expression could be upregulated by AS-IV, thus reversing the inhibition of the TGF-β/Smad signaling pathway and ultimately arresting the cell cycle in the G0/G1 phase. Moreover, Guo et al. [43] reported that the combination of NaAsO_2_ combined with AS-IV downregulates the expressions of phospho-phosphoinositide-3-kinase (*p-PI3K*), phospho-protein kinase B (*p-Akt*), the phospho-mammalian target of rapamycin (*p-mTOR*), *PI3K*, *Akt*, and *mTOR* as well as other key genes in HepG2 cells and upregulates the expression of G protein subunit gamma transducin 1 (*GNGT1*) mRNA, thus inducing cell cycle arrest in the S phase. Sun et al. [34] revealed that AS-IV induces cell cycle arrest at the G0 phase and increases the expression of *p21* both in vitro and in vivo. Lupeol independently activates CDK inhibitor 2A in Hep-2 and UPCI-SCC-131 without activating p21, thereby inhibiting the expression of Cyclin D1 and ultimately leading to tumor cell cycle arrest in the G2 phase [38]. CAG [37] negatively regulates the Janus kinase 2/Signal transducer and activator of the transcription 3 (STAT3) axis, causing GC cell cycle accumulation in the G1 phase.

Extracellular signals must navigate a specific pathway to reach the nucleus, with the mitogen-activated protein kinase (MAPK) serving as a transmitter and facilitating the transmission of cell surface signals into the nucleus. Upon receiving growth or proliferation stimuli, MAPK/extracellular signal-regulated kinase (ERK) is phosphorylated and active. After entering the nucleus, activated ERKs attach to target genes to control physiological processes, including cell division and proliferation. Notably, AS-IV [40] inhibits glioma growth in vivo and suppresses the proliferation of glioma cells by inhibiting the activity of the MAPK/ERK signaling pathway in U251. Moreover, PI3K transmits mitotic signals to target genes via Akt/mTOR to promote cell proliferation. AS-IV [43] inhibits the expression of p-PI3K, p-Akt, and p-mTOR, thereby inhibiting the proliferation of lung and liver cancer cells through the PI3K/Akt/mTOR signaling pathway. In retinoblastoma (RB) mice, treatment with lupeol [46,47] not only inhibits skin cancer by inhibiting PI3K, Akt phosphorylation, nuclear factor kappa-B (NF-κB)/p65, and IKKα activity but also inhibits the proliferation of ocular cancer cells and suppresses tumor development in vivo.

### 3.2. Regulation of Cell Death

Normal cells maintain self-renew by regulating apoptosis through gene regulation. In contrast, tumor cells proliferate indefinitely owing to reduced apoptosis [120]. Presently, induction of apoptosis represents an important mechanism for antitumor activity. Apoptosis typically occurs through two classical pathways: the endogenous pathway of mitochondrial apoptosis and the exogenous pathway of death receptors. Research has demonstrated that *A. membranaceus* saponins induce apoptosis in lung cancer, breast cancer, gastric cancer, HCC, and CEC.

#### 3.2.1. Induction of Apoptosis

The key components of the endogenous apoptotic pathway include cytochrome C (Cyt C), the caspase family, and the Bcl-2 protein family. Tumor cell apoptosis is initiated by the coordinated action of pro-apoptotic proteins and anti-apoptotic proteins [121] such as B-cell lymphoma-2 (Bcl-2) and B-cell lymphoma-extra large (Bcl-xL). AS-IV [122] induces apoptosis in NSCLC and lung adenocarcinoma cells by inhibiting the Akt/Glycogen synthase kinase 3β (GSK-3β)/β-catenin signaling pathway, inhibiting Bcl-2, and promoting the expression of Cyt C, Omi, Bax, and caspase3. In addition, oxidative stress (OS) [123] causes changes in mitochondrial membrane potential, which reduces cellular ATP, leading to apoptosis. Sun et al. [34] demonstrated that AS-IV reduces p21 expression and induces apoptosis by upregulating Bax/Bcl-2 by regulating Poly ADP-ribose polymerase (PARP) and caspase-3,-9. Moreover, CHS [51] activates caspase-3 and caspase-9 and reduces mitochondrial apoptotic peptidase activating factor (Apaf 1), inducing breast cancer cell apoptosis through the mitochondrial endogenous pathway. Lupeol [63] upregulates PARP and caspase-3 expression, thus inducing apoptosis. HDG [48] inhibits the development of head and neck cancer by inducing apoptosis in HN9-cisRHNC cells through the activation of the mitochondria-dependent endogenous system via the nuclear factor (Nrf2)/antioxidative response element (ARE) antioxidant pathway. In a previous study, uterine leiomyoma cells ULM-1 and ULM-2 isolated from patient tissues were cultured with different concentrations of AS-IV [64]. The result indicated that indoleamine 2,3-dioxygenase 1 (IDO1) may participate in the inhibition of AS-IV in ULM cells by regulating cell apoptosis. Furthermore, Kim et al. [66] observed that the levels of Nrf2, heme oxygenase-1 (HO-1), quinone oxidoreductase 1 (NQO1), and XCT are decreased, suggesting that blocking the Nrf2-ARE signaling pathway promotes apoptosis.

Endoplasmic reticulum stress (ER) [124] is a cellular protective stress response and an important mode of intracellular protein repair. Although an appropriate ER stress response is essential for cells to maintain normal physiological and biochemical functions, aberrant ER stress may lead to apoptosis. Despite its detrimental effects on the body, ER stress-based therapies are widely used in cancer treatment owing to their ability to induce apoptosis in cancer cells. In PCA cells, AS-IV-induced [59] ER stress induces apoptosis by increasing the expression of the ER stress genes binding immunoglobulin protein (*BIP*), C/EBP homologous protein (*CHOP*), and *caspase-12* mRNA. Subsequent investigations demonstrated that DS promotes c-Jun N-terminal kinase (JNK) phosphorylation, suggesting that autophagy is necessary for the apoptotic response generated by DS [93].

Another significant apoptotic mechanism in cells is the exogenous death receptor pathway, where apoptosis is initiated by death receptors allowing death signals from outside the cell to enter. HDG, DS, AMS, AST, AS-IV, and other substances can all activate the death receptor pathway. AS-IV [54] activates the factor-related apoptosis (Fas)/factor-related apoptosis ligand (FasL) signaling pathway, inducing apoptosis in 143B-homozygous mice. Additionally, AS-IV increases the chemosensitivity of osteosarcoma (OS) cells. In SK-Hep1 and Hep3B, AS-IV [55] not only increases the levels of caspase-3/-8/-9 in cells but also inhibits the expression of the anti-apoptotic proteins XIAP, MCL1, and CFLIP.

Wang et al. [41] reported that AS-III inhibits breast cancer development by promoting the apoptosis of breast cancer cells. They also speculated that the underlying mechanism is akin to that of AS-II, regulating the Akt/NF-κB signaling pathway to induce apoptosis in human leukemia cells [125]. Hu et al. also discovered that AST reduces the binding activity of NF-κB to DNA and promotes the activity of ERK1/2, thereby regulating the NF-κB/ERK signaling pathway and inducing an apoptotic cascade in HCC. Moreover, *Astragalus mongholicus* (AMs) [56] induce the activation of dendritic cells (DCs) to express Toll-like receptor 4 (TLR4) and suppress the expression of the inhibitor of NF-ĸB (IKB). Moreover, AMs inhibit the growth of tumors and enhance the lifespan of MKN45 GC mice in vivo. In p53-deficient NSCLC cells, DS [58] suppresses the normal activity of the antioxidant enzyme TrxR1 and exhibits minimal toxicity, suggesting a potential role of p53 in apoptosis.

MicroRNAs (miRNAs) play a role in regulating apoptosis by directly or indirectly targeting genes or signal transduction involved in cell death. Cui et al. [49] examined the impact and molecular mechanism of AS-IV on mice with HCC and reported that miR-150-5p controls apoptosis and tumor growth via β-catenin. AS-IV was also found to upregulate the levels of miR-150-5p, consequently facilitating the aforementioned processes. In OS cells, miR-212-3p [65] induces growth inhibition and subsequent programmed cell death. Notably, HMGA2, which is directly affected by miR-212-3p, was found to counteract these effects of miR-212-3p, whereas lupeol successfully was found to increase the expression of miR-212-3p and facilitate its ability to target HMGA2, resulting in the initiation of programmed cell death in OS cells. Li et al. [62] investigated the role of long non-coding RNA (lncRNAs) in the antitumor mechanism of AS-IV and demonstrated that increasing the expression of lncRNA-ATB counteracts the apoptosis induced by AS-IV in HCC cells. Moreover, they found that AS-IV induces apoptosis in HCC cells by reducing the expression of lncRNA-ATB, hence deactivating the IL-11/STAT3 signaling pathway. Similarly, the activation of AST results in the upregulation of facilitated programmed cell death [57].

#### 3.2.2. Regulation of Autophagy

Autophagy plays a dual role in tumor cells, with drug-induced autophagy often leading to two contrasting outcomes [126]. First, autophagy protects tumor cells and mitigates the detrimental impacts of the surrounding environment. Second, it triggers an increase in the autophagic activity of tumor cells, leading to the initiation of type II programmed cell death. This dual nature of autophagy presents two distinct approaches for preventing and treating tumors: inhibiting autophagy to enhance the efficacy of anti-cancer treatments or stimulating autophagy to induce tumor cell death. Autophagy-associated (Atg) proteins [127], including Vps34, Beclin1, the Vps34 complex of Atg14 and Vps15 (p150), and ULK1, play pivotal roles in the regulation of the autophagic process.

Research has demonstrated that AS-IV and AS-II can suppress the process of autophagy in cancer cells. Furthermore, AS-IV can either enhance or suppress cellular autophagy by controlling Ag (antigen) proteins. AS-II [87] sensitizes Bel-7402/FU cells by decreasing the levels of LC3-phosphatidylethanolamine conjugate (LC3-II) and Beclin-1 expression, inhibiting autophagy. A comprehensive investigation revealed that the process of autophagy in HCC is suppressed by modulating the MAPK-mTOR signaling pathway. Astragalus–scorpion [88] medicines have been found to target 163 proteins in the PCA, primarily associated with the PI3K/Akt signaling pathway. Moreover, Astragalus–scorpion can hinder the development of PCA, enhance the production of LC3-II and Beclin1 proteins, and suppress the production of P62 and PI3K-AKt pathway proteins. Notably, these effects were reversed upon GDPD4-2 silencing, indicating that GDPD4-2 plays a role in the mechanism by which Astragalus–scorpion targets PCA by regulating the PI3K/AKt/mTOR pathway.

Yu et al. [92] employed a combination of molecular docking and network pharmacology, identifying the Ras/Raf/MEK/ERK signaling pathway as a potential target for AST. Subsequent investigations demonstrated that the act of removing phosphate groups from MEK and ERK, known as functional dephosphorylation, hindered the Ras/Raf/MEK/ERK signaling pathway. This pathway ultimately converges at mTOR and triggers the process of autophagy. In podocytes stimulated by glucose, AS-IV [73] increases autophagy by decreasing the acetylation of the NF-κB p65 subunit and enhancing the production of Beclin 1. In RAW 264.7 cells exposed to cigarette smoking extract, AS-IV [90] triggers autophagy by blocking the TLR4/NF-κB signaling pathway. In SW962 cells, AS-IV [39] induces autophagy, resulting in a considerable elevation of Beclin-1 and LC3-II levels, while causing a drop in p62 protein levels. AS-IV also regulates specific proteins that impact the growth and structure of CCA cells. Furthermore, SiHa and HeLa cells have been discovered to contain two autophagy-related proteins. AS-IV [89] has been found to stimulate and strengthen autophagy in CCA cells by specifically targeting and regulating autophagy proteins, namely DCP1A and TMSB4X. This leads to an increase in the expression of LC3I/II, autophagy-related 7 (Atg7), and Atg12 in HeLa and SiHa cells.

In addition, DS [45] suppresses the growth and cell proliferation of liver cancer both in vitro and in vivo. Further studies demonstrated that DS effectively stimulates the generation of reactive oxygen species (ROS) and the conversion and accumulation of LC3-II, providing evidence that DS can trigger autophagy through a process that relies on ROS. Moreover, AS-IV [60] sensitizes NSCLC cells by suppressing the expression of glucose-regulating protein 78 (GRP78) and Beclin1, decreasing ER stress and autophagy.

### 3.3. Inhibition of Tumor Cell Metastasis

Cell migration and invasion, the processes by which cells move from their original site to a distant organ, are crucial for cancer recurrence. Epithelial–mesenchymal transition (EMT) is a significant mechanism in cancer metastasis [128], facilitating enhanced invasion and destruction of the extracellular matrix (ECM) by tumor cells. The levels of E-cadherin, cytokeratin, N-cadherin, and vimentin expression serve as specific molecules for detecting EMT [129]. Notably, Astragalus saponins, specifically AS-IV and SsaI, can hinder EMT and consequently impede tumor growth.

The EMT is regulated by the Wnt/β-cadherin signaling pathway. Additionally, AS-IV significantly reduces the expression of Wnt, β-catenin, and TCF-4. These findings suggest that AS-IV effectively inhibits the migration and invasion of HCC cells by regulating the Wnt/β-catenin pathway. In M2 macrophages activated by interleukin 4 (IL-4) and IL-13, AS-IV [97] suppresses the TGF-β/Smad2/3 signaling pathway, resulting in the suppression of CCA cell migration and EMT. Furthermore, research has demonstrated [83] that AS-IV effectively suppresses the TGF-β/Akt/Foxo1 signaling cascade in M2 macrophages. AS-IV has been discovered to inhibit the migratory and invasive activities of breast cancer cells generated by M2 macrophages. Previous research has demonstrated [67] that TGF-β1 pretreatment in U251 cells results in the excessive production of β-catenin proteins, which in turn reduces the expression of proteins such as E-cadherin. However, AS-IV can reverse these effects caused by TGF-β1, providing evidence of the essential role of TGF-β1 in the transformation of E-cadherin into N-cadherin. Moreover, AS-IV can effectively hinder the migration and infiltration of U251 cells by reducing the TGF-β1-induced EMT-associated Wnt/β-catenin signaling pathway. Zhao et al. [130] investigated the mechanism by which AS-IV inhibits the spread of invasive metastasis in VSCC. They found that treatment with TGF-β1 upregulates the expression of N-cadherin and vimentin but downregulates that of E-cadherin. Moreover, treatment with AS-IV results in the downregulation of p-focal adhesion kinase (FAK), p-AKt, matrix metalloproteinase 2 (MMP-2), and matrix metalloproteinase 2 (MMP-9) expression. These findings suggest that AS-IV blocks the TGF-β1/FAK/Akt signaling pathway, leading to the inhibition of cell motility and invasion. In HCC and NSCLC cells, AS-IV [50,68] suppresses the formation of EMTs by specifically targeting the Akt/GSK-3β/β-catenin signaling pathway, increasing the expression of E-cadherin and decreasing the expression of N-cadherin. AS-IV has also been shown to hinder the migration and infiltration of cells and suppress the progression of HCC and NSCLC by blocking the stimulation of the Akt/GSK-3β/β-catenin pathway.

The PI3K/Akt/NF-κB pathway is frequently involved in inhibiting tumor cell EMT. AS-IV suppresses the phosphorylation of PI3K, Akt, and NF-κB, impacting the migratory and invasive capabilities of NSCLC cells. AS-IV [70] reduces the expression of E-cadherin, MMP-2, and MMP-9 and suppresses the movement and infiltration of A549 cells by blocking the PKC-α-ERK1/2-NF-κB signaling pathway. Zhang et al. [71] discovered that AS-IV exhibits a notable therapeutic impact on MCF-7 transplanted tumor-bearing mice. Specifically, AS-IV effectively decreases the levels of MMP-2 and MMP-9 proteins and dramatically lowers the expression of PI3K, Akt, and mTOR. These findings indicate that AS-IV effectively impedes the movement and infiltration of thymic cancer cells by reducing the PI3K/Akt/mTOR signaling pathway. Lupeol [69] reduces the concentrations of urokinase plasminogen activator (uPA), MMP-2, MMP-9, and N-cadherin while increasing that of VE-cadherin in OS cells. Lupeol also induces a considerable reduction in the expression levels of PI3K, p-AKt, and β-catenin while simultaneously increasing that of GSK-3β. This finding indicates that lupeol effectively suppresses β-catenin, thereby suppressing the activity of MMP-2 and MMP-9. These signaling pathways, namely PI3K/Akt/GSK-3β and p38/MAPK, eventually hinder the migration and invasion of OS cells. Moreover, AS-IV [73] suppresses the generation of glucose-induced EMT by regulating the silent information regulator 1 (SIRT1)/NF-κB pathway and inhibiting reducing it.

In addition, miRNA plays a role in suppressing the EMT of cancer cells. CREB 1 [76], which is a key target of miR-134 in SW-480 cells, greatly promotes the migration and invasion of SW-480 cells. AS-IV increases the expression of miR-134 and decreases that of *CREB 1* mRNA and protein, resulting in the suppression of colon cancer cell motility and invasion. Furthermore, a previous study suggested that AS-IV could potentially hinder the processes of EMT and angiogenesis in GC by increasing the expression of programmed cell death 1 ligand 1 (PD-L1), a protein that is controlled by miR-195-5p [75]. EIF4A1 [74] is a specific target of miR-489-3P, and circDLST has been reported to block miR-489-3p to control EIF4A1, resulting in increased cell motility and invasion. Nevertheless, AS-IV can suppress the expression of circDLST and EIF4A1, indicating that AS-IV hinders the migratory and invasive capabilities of GC cells via modulating the circDLST/miR489-3p/EIF4A1 pathway. Notably, AS-IV [84] significantly upregulates MCM5 in both lung cancer cells and tissues; MCM5 and HDAC1 facilitate the proliferation and migration of lung cancer cells, whereas AS-IV restores the expression of E-cadherin, hence suppressing lung cancer cell activity. Moreover, AS-IV decreases the expression of circ0001615 and LASP1 while increasing that of miR-873-5P to suppress the development of CRC [131].

In cancer cells, lncRNAs are abundantly expressed, and their expression is closely related to cancer development, playing a role in regulating tumor cell migration. In hepatoma cells, lncRNA-ATB has been shown to promote the development of EMT [62]. Notably, in SMMC7721 and Huh-7 cells, AS-IV suppresses the EMT and migration by reducing the expression of IncRNA-ATB. In contrast, the upregulation of lncRNA-ATB counteracts the effects of AS-IV on the migration of liver cells, EMT, programmed cell death, cell survival, and the transmission of IL-11/STAT3 signals. Finally, AS-IV suppresses the migratory and survival abilities of HCC cells by reducing the expression of lncRNA-ATB. Hu et al. [77] found that AS-IV upregulates TRHDE-AS1 in vitro and in vivo, contributing to the downregulation of MMP-2 and MMP-9 expression, thereby inhibiting breast cancer cell migration and invasion.

The ECM is primarily regulated by matrix-degrading enzymes, including MMP and MAPK. Wang et al. [82] discovered that the decrease in MMP-2 and MMP-9 expression and inhibition of invasion in a laboratory setting may be attributed to the prevention of p38 MAPK activation. In addition, the administration of AS-IV [80] to mice with transplanted U251 and MDA-MB-231 tumors results in reduced levels of MMP-2 and MMP-9. Subsequent research has revealed that the suppression of cell migration in glioma and breast cancer occurs through the interaction of MAPK/ERK and Vav guanine nucleotide exchange factor-3 genes. ECM functions to provide mechanical support for cells and tissues. The process of carcinogenesis involves the aberrant restructuring of the ECM, affecting the migration and infiltration of cells. A previous study reported [79] that while SsaI enhances the attachment of melanoma cells to ECM proteins, it has no impact on the invasion of melanoma cells, indicating that SsaI may have altered ganglioside GM3 (NeuAca2-3Galb1-4Glcb1-10Cer). Moreover, SsaI [78] suppresses the expression of α2,3-linked sialic acids, resulting in a decrease in B16F10 migration. Furthermore, it decreases the production of ST3Gal IV, thereby improving the ECM. Notably, DS [81] exerts anti-metastatic effects by reducing chemotaxis and PCA migration while simultaneously enhancing cell adherence to fibronectin and collagen matrix.

### 3.4. Inhibition of Angiogenesis

Tumor angiogenesis is critical for tumor growth and metastasis. By promoting angiogenesis, i.e., the formation of new blood vessels, tumors can access sufficient resources and oxygen to sustain the nutritional needs for rapid growth [132]. In addition, the atypical configuration and operation of neovascularization facilitate the infiltration of tumor cells into adjacent tissues from the original tumor location, resulting in the development of distant metastases. Vascular endothelial growth factors (VEGFs) play a crucial role in promoting tumor angiogenesis. In addition to VEGF, many cytokines and signaling pathways also play a role in controlling tumor angiogenesis.

In SGC-7901 cells, AS-IV [133] suppresses cell growth by decreasing the activity of cyclooxygenase-2, resulting in the reduction of prostaglandin E2 production and the downregulation of VEGF, ultimately leading to a decrease in tumor growth. Furthermore, research using A549 and U251 cells also observed a reduction in VEGF expression. In vascular endothelial cells, AS-III [85] induces transcatheter arterial chemoembolization and activation of the epidermal growth factor receptor (EGFR), subsequently leading to the activation of ERK1/2 and AKt as well as the activation of anti-inflammatory and growth factor signaling. In mice bearing H22 transplanted tumors, the protein expression levels of VEGF, MMP-2, MMP-9, aquaporin-1, and platelet endothelial cell adhesion molecule-1 (CD31) decreased following treatment with AS-IV. This result indicates that AS-IV inhibits the development of H22 ascites-associated HCC by inhibiting angiogenesis and the expression of proteins involved in cell migration and water transport. In H1299 cells, lupeol exhibits a strong attraction to VEGFR2 and suppresses the production of chemokines, hence impeding the progression of lung cancer [134].

### 3.5. Enhancement of Cellular Immunity

#### 3.5.1. Enhancement of Cellular Nonspecific Immunity

Tumor tissues are invaded by numerous inflammatory cells [135] such as tumor-associated macrophages (TAMs), lymphocytes, and mast cells, with TAMs being the most prevalent. TAMs can be phenotypically identified as polarized towards the M2 phenotype, which promotes tumor advancement. Macrophage colony-stimulating factor 1 (CSF1), IL-4, IL-10, TGF-β, and IL-13 promote the differentiation of macrophages into the M2 subgroup.

In M2 macrophages, AS-IV [95] suppresses the activation of AMP-activated protein kinase (AMPKα), therefore blocking the AMPK signaling pathway. It also reduces the levels of IL-10 and TGF-β, preventing the polarization of M2 macrophages caused by IL-13 and IL-4. Shen et al. examined the correlation between the antitumor properties of AS-IV and macrophages and demonstrated reduced levels of CD10, IL-206, TGF-β, and CD2, suggesting that AS-IV suppresses macrophage polarization. Moreover, the protein expression levels of p-Smad3 and p-Smad2 are markedly reduced in CCA cells cultured in conditioned media, whereas the administration of TGF-β reversed these effects [97]. These results indicate that AS-IV suppresses the polarization of M2 macrophages, promoting EMT in CCA cells by blocking the TGF-β/Smad3 signaling pathway.

In addition, AS-IV [96] could potentially protect against M2 macrophage-induced ovarian cancer by suppressing the HmgB1-TLR4 signaling pathway. In macrophages co-cultured with ovarian cancer, AS-IV suppresses the elevated levels of HmgB1 and TLR4, further supporting the notion that HmgB1 can promote M2 macrophages, whereas AS-IV has the opposite effect.

Natural killer cells (NK cells) play a crucial role in eliminating abnormal cells in conditions such as aging, viral infections, and tumors. Unlike some immune cells, NK cells do not require specific instructions from the immune system to recognize and attach their targets. Wu et al. [98] indicates that at optimal doses, lupeol can enhance the growth of NK cells; suppress the growth of GC cell lines BGC823, N87, and HGC27; and enhance the ability of NK cells to eliminate GC cells. Moreover, AS-III [72] markedly enhances the expression of the T-bet transcription factor and upregulates the expression of IFN-γ and IL-12 both in vivo and in vitro, enhancing the anti-colorectal cancer impact of NK cells.

AS-IV has been demonstrated to enhance the immune response by controlling the production of cytokines, nitric oxide (NO), and mRNA and/or protein expression involved in cell cycle regulation. This effect is particularly evident in the regulation of IL-1β, IL-6, and tumor necrosis factor α (TNF-α), and it is mediated by the NF-κB/MAPK pathway. AS-IV [99], which can inhibit cell proliferation, has been shown to regulate the expression levels of Cyclins D1, CDK4, and CDK6 in the host organism, resulting in the release of CDS, specifically CD40 and CD86. AS-IV can also suppress the activity of gastric cancer-associated fibroblasts (GCAFs) [101], upregulating miR-214 and down regulating miR-301a expression. Moreover, AS-IV inhibits the upregulation of important proteins such as SRY-box2 (SOX2) and Nanog homeobox, known to induce pluripotency in somatic cells, by GCAFs. In addition, AS-IV decreases the levels of macrophage-CSF and increases that of TIMP-2. CD147 is essential for the function of monocarboxylate transporter 1 (MCT1) and MCT4 in GC. Zhang et al. [100] reported that AS-IV had a beneficial effect in reducing precancerous lesions of gastric carcinoma by inhibiting glycolysis through the regulation of the p53/miRNA-34a/LDHA and p53/TIGAR pathways. Additionally, AS-IV could restore the levels of MCT1/4, CD147, and HIF-1α, providing evidence that AS-IV inhibits the progression of GC. However, this finding requires validation in clinical studies.

#### 3.5.2. Enhancement of Specific Immunity

Cytotoxic T cells [136], also known as cytotoxic lymphocytes (CTL), express cell surface markers CD3+, CD4+, and CD8+. These cells primarily target tumor cells that display specific antigens and class I MHC molecules, resulting in their destruction. Throughout the development of a tumor, the tumor microenvironment (TME) hinders the functioning of the immune system, resulting in the deterioration of CTL activity and the corresponding immune evasion.

The combination of AS-IV [103] and oxidized picloram promotes the invasion of CD4 and CD8 T cells, effectively inhibiting the progression of triple-negative breast cancer. AS-IV [94] exerts an immunostimulatory impact by suppressing the production of *IDO* mRNA and protein in lung cancer cells, resulting in a decrease in the number of regulatory T lymphocytes and an increase in CTL activity, culminating in an anti-lung cancer effect. AS-IV [137] additionally stimulates the production of IL-1β, IL-6, and TNF-α at both the protein and mRNA levels. It also upregulated the levels of *NO* and *iNOS* mRNA, consequently augmenting the immunological function of RAW264.7 and TAW264.7 cells. Treatment with IL-1, IL-2, and PMA [95] induces the differentiation of human THP-4 monocytes into M13 macrophages. Similarly, in a previous study, IL-1 and lung cancer cell lines A2 and H2 were cultivated in a conditioned medium with M549 macrophages (M1299-CM) to induce their development. The results demonstrated that AS-IV effectively suppresses the invasion, migration, and formation of new blood vessels in these. This effect may be attributed to the suppression of AMPKα activation in M2 macrophages by AS-IV. Ma et al. [102] administered AS-IV to mice with Huh-7 transplanted tumors and demonstrated that AS-IV effectively decreases the expression of PD-L1. Notably, the immunosuppression mediated by AS-IV involves the regulation of the miR-135b-5p/CNDP1 axis as its major mechanism. Furthermore, the combination of AS-IV with the checkpoint inhibitor ApD-1 [35] can exert a synergistic effect in treating CRC, this effect is achieved by preventing tumor development and enhancing the formation of T cell fibrillation. AS-IV serves a distinct function in several physiological processes. B7-H3 can decrease the T-assisted type 1 immune response, which leads to the inhibition of CD4+T cell activation and cytokine generation, thus facilitating the evasion of cancer cells from the immune system. In SW620 and HCT116 cell lines, AS-IV [44] upregulates the expression of miR-29c, which in turn targets the binding of B7-H3, leading to a decrease in the levels of B7-H3 expression in CRC.

### 3.6. Combination Therapy

#### 3.6.1. Improvement of Multidrug Resistance

Prolonged chemotherapy treatment results in the emergence of multidrug resistance (MDR). The crucial aspect of treatment in this scenario is to suppress the mechanisms of MDR, which involve drug efflux, drug absorption by transporter proteins, stimulation of drug metabolism, and drug target mutation [138].

AS-IV [111] can effectively overcome the resistance of Bel-7402/FU cells to 5-FU by blocking the translation process of p-glycoprotein, resulting in lower expression of p-glycoprotein. This results in a greater accumulation of 5-FU in Bel-7402/FU cells, ultimately enhancing the sensitivity of these cells to 5-FU. Wang et al. [112] discovered that the MDR of Bel-7402/FU cells is associated with the JNK pathway and confirmed that AS-IV suppresses the expression of MDR1 to hinder the JNK pathway, hence alleviating MDR. They additionally indicated that the expression of MDR1 is regulated by the JNK/c-Jun/AP-1 pathway.

Resistance to adriamycin (DOX) is highly common in cancer treatment. Notably, AS-II [87] efficiently suppresses the expression of the MDR 1 gene, hence reversing MDR and inhibiting the accumulation of Rh123 through the efflux assay. Further research into this process revealed that AS-II stimulates the activation of MAPK while suppressing the phosphorylation levels of ERK 1/2, JNK, and p38. Nevertheless, the full comprehension of the pharmacological impact of AS-IV remains incomplete. The compounds HQ [114], AS-IV, calyxin (CS), or metanephrines (FMNT) have been shown to upregulate the expression of Nrf 2, p-glycoprotein, and BCRP, resulting in a considerable increase in cellular exocytosis. Hence, it is imperative to focus on the interplay of herbs such as HQ, AS-IV, and CS when used alongside other medications.

#### 3.6.2. Increase of Sensitivity to Anti-Cancer Agents

Presently, the concurrent administration of *A. membranaceus* saponins and chemical medications has been demonstrated to alleviate the resistance of tumor cells to chemotherapy treatments. For example, *A. membranaceus* saponins enhance the cytotoxic effects of chemical medicines on tumor cells by facilitating programmed cell death, inhibiting the migration of tumor cells, and blocking the process of autophagy [139].

Upon administering a combination of AS-IV [54] and cisplatin to mice with 143B graft tumors, AS-IV elevates the expression level of Fas/FasL, hence promoting the apoptosis of OS cells. Chen et al. [52] examined the therapeutic efficacy of lupeol on LoVo mice that were resistant to oxaliplatin and demonstrated that lupeol effectively suppresses tumor growth and triggers programmed cell death by decreasing the expression of ABCG2 and activating the ER stress pathway. Li et al. [61] isolated AS-IV and chlorogenic acid compounds from traditional Chinese medicine to synthesize RLT-03 and investigated this compound for its anti-cancer properties against breast cancer. They demonstrated that the administration of RLT-03 to EMT6 xenograft mice suppresses the expression of receptor tyrosine kinase (RTK) and inflammatory markers while also triggering death in breast cancer cells.

In rat microglia, LOC102555978 interacts with miR-3584-5p to enhance NLRP3-mediated cellular pyroptosis [140], whereas AS-IV hinders LOC102555978, thereby hindering the effect of LOC102555978. A previous study investigated the combination of AS-IV [105] and carboplatin in in vitro and in vivo experiments using PC-3 transplanted tumor-bearing mice and demonstrated that this combination exhibited a potent effect in suppressing tumor growth. This effect was mainly attributed to the ability of AS-IV to regulate the Akt/NF-κB pathway, inhibiting carboplatin-induced EMT. According to Ye et al. [76], miR-134 exhibits a direct association with the protein CREB1, which in turn is linked to drug resistance. Furthermore, AS-IV or miR-134 has a substantial inhibitory effect on the EMT of colon cancer cells and enhances the responsiveness of CRC cells to oxaliplatin.

In addition, AS-II [141] increases the expression of LC3II and decreases that of p62, therefore promoting the process of autophagy. Furthermore, AS-II enhances the responsiveness of ovarian cancer cells to cisplatin, likely via the Akt/mTOR signaling pathway. Cisplatin [60] triggers ER stress and autophagy in NSCLC cells, specifically A549^cis^ and H1299^cis^, whereas AS-IV prevents these effects, enhancing the sensitivity of these tumor cells to cisplatin. Wang et al. [104] administered a combination of paclitaxel and HDG to mice with NCI-H1299 transplanted tumors and demonstrated that HDG enhances OS and the effectiveness of paclitaxel in inhibiting tumor growth. Liu et al. [53] examined the impact of co-administering HDG and cisplatin on Ncl-H6 lung cancer in mice and demonstrated that HDG enhances the responsiveness of cancer cells to cisplatin, resulting in increased caspase-3 and PARP levels as well as increased ROS levels in lung cancer cells.

Moreover, the activation of certain pathways and genes plays a crucial role in the treatment of MDR conditions and is linked to heightened drug responsiveness. Combining AS-IV [106] with cisplatin effectively hinders the growth of A549 and HCL-H1299 cells by suppressing the levels of *B7-H3* mRNA and protein expression. This suppression leads to increased sensitivity of NSCLC cells to cisplatin. Multiple investigations have substantiated the involvement of NOTCH3 in cell proliferation, migration, and death. For example, the overexpression of *NOTCH3* [107] could enhance the growth of colon cancer cells HCT 116 and SW 480, whereas the combination of AS-IV and cisplatin dramatically enhances the susceptibility of colon cancer cells to cisplatin. AS-IV [108] increases the expression of the inhibitory gene *SIRT6* in A549 cells, enhancing the sensitivity to gefitinib in NSCLC. In their research, Zheng et al. [109] investigated the impact of AS-IV on enhancing the chemical sensitivity of breast cancer cells to paclitaxel. The primary focus of their investigation was to examine the role of AS-IV in mediating the effects of caveolin-1 (CAV-1). They accomplished this by utilizing both a *CAV-1* overexpression plasmid and siRNA and demonstrated that AS-IV suppresses the expression of CAV-1 and stimulates the endothelial nitric oxide synthase/nitric oxide pathway, thereby enhancing the chemical responsiveness of breast cancer to paclitaxel. The combination of CAG and paclitaxel was also investigated, and CAG was found to exhibit cytotoxic effects only at a level of 5 μM, with no significant impact on the sensitivity to paclitaxel at higher concentrations [37]. In contrast, the combination regimen was shown to decrease the continuous activation of STAT3 in cells compared to monotherapy. This finding holds therapeutic significance, particularly as patients diagnosed with stomach cancer frequently encounter significant adverse effects and develop resistance to chemotherapy. Thus, the combination of CAG and paclitaxel can induce a substantial apoptotic impact by considerably attenuating the STAT3 axis. Furthermore, the medication combination has the potential to mitigate the adverse effects of paclitaxel.

#### 3.6.3. Amelioration of Side Effects and Toxicity

Anti-cancer drugs especially chemotherapeutic agents have been widely reported to induce side effects and toxicity to organs and tissues, such as cisplatin causing acute liver injury and nephrotoxicity and DOX inducing cardiomyopathy [116,142,143]. Numerous studies have shown that *A. membranaceus* saponins ameliorate the potential toxic side effects through suppression of oxidative stress, inflammation, and other related pathways. In a previous study on the administration of a combination of AS-IV [110] and cisplatin to H22 transplant tumor mice, AS-IV was found to suppress the expression of MRP 2 and decrease that of chromium and urea nitrogen, alleviating the nephrotoxicity induced by cisplatin. Furthermore, AS-IV mitigates the destruction of iron cells caused by cisplatin treatment by enhancing the expression of PPARα, which is suppressed by cisplatin [142].

NADPH oxidase (NOX) is a group of enzyme proteins located in the plasma membrane. It is composed of seven components, namely DUOX1, DUOX2, NOX1, NOX2, NOX3, NOX4, and NOX5. NOX2 and NOX4, which are part of the NOX family, are present in the heart and play a role in increasing ROS levels within the cells. OS has been recognized as a primary factor contributing to the development of DOX-induced cardiomyopathy. Administration of DOX has been demonstrated to elevate the levels of NOX2 and NOX4 in the hearts of animals [113], consequently leading to an increase in cardiomyopathy mediated by ROS. In contrast, AS-IV significantly mitigates the development of heart muscle disease caused by DOX, decreases the OS caused by NOX2 and NOX4, and alleviates the negative effects of doxorubicin treatment. Therefore, AS-IV appears to be a promising addition to chemotherapy. For example, it can alleviate the adverse effects induced by sunitinib on the heart by blocking the activity of COUP transcription factors [144].

In addition, Luo et al. [115] demonstrated that AS-IV improves adriamycin-induced myocardial iron death by blocking the Nrf2 signaling pathway. Xie et al. [117] employed cisplatin-induced acute renal damage in mice and reported that the administration of HDG results in the inhibition of Axin2 and its downstream β-catenin signaling. This, in turn, improves the condition of cisplatin-induced kidney injury and inflammation. Nguedia et al. [81] induced the development of breast tumors in rats by administering DMBA and then treating them with DS. Their research revealed that DS exhibits antioxidant properties and also increases catalase activity. In addition, AS-IV [48] effectively suppresses liver fibrosis by modulating the activity of pSmad3C/3L and enhancing Nrf2 phosphorylation, therefore impeding the progression of liver cancer. Gong et al. [143] demonstrated that AS-IV improves liver fibrosis and retards the progression of hepatocellular carcinoma by facilitating communications between the pSmad3C/3L and Nrf2/HO-1 signaling pathways. Specifically, they highlighted the impact of Nrf2/HO-1 on the antitumor action of AS-IV. Recent research has discovered that in SD mice subjected to cisplatin treatment [91], AS-IV promotes autophagy, reduces the expression of pyrin-containing structural domain (NLRP3), and offers considerable protection against liver and kidney harm caused by cisplatin. Moreover, AS-IV suppresses cardiomyocyte pyroptosis by increasing the expression of SIRT1 and reducing the activation of NLRP3, hence functioning as a pre-protective agent [116].

## 4. Discussion and Perspectives

In recent years, an increasing number of studies have demonstrated that *A. membranaceus* saponins provide cardiovascular protection [144], hepatoprotective effects [142], anti-diabetic properties [145], immune enhancement [146], and anti-inflammatory benefits [147]. In particular, *A. membranaceus* saponins can inhibit the development of various types of cancers, including lung, breast, and CRCs [148], mainly owing to their properties of inducing apoptosis and inhibiting tumor cell migration and invasion. In addition, *A. membranaceus* saponins can significantly reduce the toxic side effects of some chemical drugs by combining them with other chemical drugs [149,150].

However, significant strides are yet to be made before *A. membranaceus* saponin can be developed into a marketed drug, and studies targeting *A. membranaceus* saponin are still limited. AS-IV have achieved most attention among the *A. membranaceus* saponins. Most *A. membranaceus* saponins possess a cycloartane-type triterpenoid skeleton named cycloastragenol, modified by glucosylation at the C3/C6/C25-OH and/or xylosylation at C3-OH. Thereinto, AS-IV has been documented as one of the main active ingredients of *A. membranaceus* and is used as the main marker for the quality control of *A. membranaceus* in the Chinese Pharmacopeia (2020 version). Chemical compositions analysis also indicated that the relative content of AS-IV was higher than most of the other saponins. Several comparative studies indicated that AS-IV exhibited superior efficacy compared with the other astragalosides. Thus, previous studies were mainly focused on the pharmacological effects and mechanisms of AS-IV, while the other saponins have been poorly investigated [151,152]. Otherwise, the structure–activity relationship of the *A. membranaceus* saponins is still unclear to date. In consideration of the low solubility and pharmacokinetic disadvantages after oral administration of these saponins, structure modification based on the better understanding of the structure–activity relationship is highly valued.

Despite the discovery of some signaling pathways related to the antitumor effect of *A. membranaceus* saponin, the direct targets and specific molecular networks still require further investigations using transcriptomics, proteomics, and target fishing [153,154]. Moreover, considerable variability exists in the doses of drugs administered in the various trials. Therefore, extensive pharmacokinetic and clinical studies are required to confirm the safety window and effective dose [155,156]. Finally, future research should focus on the advancement of the development of drugs based on *A. membranaceus* saponin. This could involve designing the dosage form according to the scientific properties and specific targets of *A. membranaceus* saponins. In recent years, new drug delivery systems for Astragalosides have been emerging [157,158], such as nanocrystal formulation [159], along with new formulations combined with other drugs [160]. In addition, structural modification of active ingredients can be pursued to achieve synergistic effects and reduce toxicity [161] or to further discover new lead compounds.

## 5. Conclusions

The anti-cancer effects of *A. membranaceus* saponins and their mechanisms of action have been widely investigated in the past decades. Previous in vitro and in vivo experimental data indicate that *A. membranaceus* saponins represented by AS-IV exhibit significant anti-cancer effects through multiple mechanisms, especially in inhibiting tumor cell proliferation, migration, and invasion and inducing apoptosis, inhibiting metastasis and angiogenesis, enhancing cellular immunity, etc. This review systematically compiles the research progress on the anti-cancer potential of *A. membranaceus* saponins, and future perspectives are proposed. Further mechanistic insights and clinical studies will help promote the development of *A. membranaceus* saponins as an effective drug against cancer.

## Figures and Tables

**Table 1:** Anti-cancer effects and mechanisms of *A. membranaceus* saponins.

Pharmacological Activity	Components	Models	Administration Design	Mechanisms	Refs.
Inhibition of cell proliferation	AS-IV	HT29, SW480, BALB/c mice	25, 50 μM; 24 h; 20 mg/kg; 30 days; i.g.	p21, Cyt C, and Omi↑	[34]
	AS-IV	CT26	10, 50, and 100 nM; 48 h	B7-H3, Cyclin D1, and CDK4↓	[35]
	AST	HT29	94 μM; 24, 48, and 72 h;	mTOR/NF-κB pathway↓; PTEN↑	[36]
	CAG	SNU1, SNU16	10, 30, and 50 μM; 8 h SNU16: IC_50_ 5 μM	p-STAT3, STAT3, and JAK1/2↓; caspase-3 and PARP cleavage↑	[37]
	Lupeol	HEp2, UPCI	50 μM; 72 h; HEp2: IC_50_ 53.51 μM UPCI: IC_50_ 52.44 μM	p53 and p16↑; Cyclin D1↓	[38]
	AS-IV	SW962	255 μM; 24 h	Cyclin D1↓; Bax, cleaved caspase-3, p21, and p53↑	[39]
	AS-IV	U251	50, 75, and 100 μM; 24 h	Ki67, PCNA, MMP-2, MMP-9, and MAPK/ERK pathway↓	[40]
	AS-III	MDA-MB-231, nude mice	13, 65, and 130 nM; 24, 48 h; 10, 20 mg/kg; 30 days; i.p.	mTOR/NF-κB pathway↓	[41]
	AS-IV	A549	125 μM; 24 h; A549: IC_50_ 32.2 μM	p-PI3K, p-AKt, and p-mTOR↓	[42]
	AS-IV, NaAsO_2_	HepG2	4 μM NaAsO_2_, 1 μM AS-IV; 48 or 72 h	PI3K/AKt/mTOR pathway↓	[43]
	AS-IV	SW620, HT116	0, 6, 12, 25, 62, and 126 nM; 24, 48, and 72 h	miR-29c↑; B7-H3 and NF- κB↓	[44]
	DS	MCF7, AGS, MGC803, Murine H22 hepatoma allograft model	5, 25, 50, and 100 μM; 48 h; MCG803: IC_50_ 19.96 μM, BGC823: IC_50_ 3.13 μM, AGS: IC_50_ 24.19 μM, MCF-7: IC_50_ 16.95 μM 0.5, 2.5 mg/kg; 7 days; i.g.	ROS production and LC3-II↑	[45]
	Lupeol	Y79, WERI-Rb-1	10, 20, and 40 μM; 24 h; Y79: IC_50_ 47.1 μM, WERI-Rb-1: IC_50_ 26.7 μM	Bax/Bcl-2 and LC3 II↑; Ki67, Bcl-2, and PI3K/AKt/mTOR pathway↓	[46]
	Lupeol	CD-1 mice	12, 24 mM; 30 min	iNOS↑; COX-2, and PI3K/Akt pathway↓	[47]
	AS-IV	HSC-T6, HepG2, C57BL/6J	5, 10, and 20 μM; 24 h; 20, 40, and 80 mg/kg; 20 weeks; i.g.	Nrf2/HO-1 and p-Smad3C/p21 pathway↑; p-Smad3L/c-Myc pathway↓	[48]
	AS-IV	SMMC7721, Huh7	6, 12, 25, 50, 100, 125, 150, 225, and 255 μM; 24 h; 20 μg/mL; 24 h	miR-1505p↑; β-catenin and CTNNB1↓	[49]
Induction of apoptosis	AS-IV	HCC827, A549, NCI-H1299	15 and 30 nM; 48 h	cleavage of caspase-3↑; Akt/GSK-3β/β-catenin pathway↓	[50]
	AS-IV	HT29, SW480, BALB/c nu/nu mice	12, 25, and 50 μM; 24 h; 20 mg/kg; 30 days; i.g.	Cyt C, Omi, and p21↑	[34]
	AS-III	MCF7, MDA-MB-231, nude mice	13, 65, and 130 nM; 24, 48 h; 10, 20 mg/kg; every two days, 30 days; i.p.	mTOR/NF-κB pathway↓	[41]
	HDG	MCF7, MDA-MB-231	0.2, 0.8, 4.2, and 21.2 μM; 24 h;	caspase-3 and caspase-9↑; Apaf-1 and Cyt C↓	[51]
	Lupeol	Oxaliplatin- R LoVo, nude mice	0, 20, 50, 100, and 150 μM; 24 h; LoVo: IC_50_ 15 μM 150 mM; every two days, 12 days; i.p.	ER stress↑; ABCG2↓	[52]
	HDG	LoVo cells	1.0, 2.0 μM; 24, 48 h; LoVo: IC_50_ 1.39 μM at 24 h and 1.17 μM at 48 h	Bax, caspase-3, caspase-9, Bcl-2, procaspase-9, procaspase-3, and PARP↑	[53]
	AS-IV	MG63, 143B, BALB/c nu/nu mice	20, 40, 60, 80, and 100 μM; 48 h; 20 mg/kg; 28 days; i.g.	cleaved caspase-8, cleaved caspase-3, cleaved PARP, Fas, and FasL expression↑	[54]
	AS-IV	SK-Hep1, Hep3B	0, 200, and 400 μM; 48 h	cleavage of caspase-3, caspase-8, and caspase-9↑; XIAP, MCL1, and CFLIP↓	[55]
	AS-IV	HT29	75 μM; 1 h	PTEN↑; reduce the binding activity of NF-κB to DNA, activate the activity of ERK 1/2	[36]
	AMs	MKN45, C57/BL female mouse	76–102 nM; 24 h; 250 mg/kg; 6 days; i.p.	TLR4 and CD11c↑; IκB-αby↓	[56]
	AST	MGC803, MKN45, HCT116, MCF7, HepG2, HT29, Caco2, DLD1	94, 125 μM; 24 h, 48 h; HCT116: IC_50_ 94 μM	NAG-1, Egr-1, and PARP cleavage↑; PI3K/Akt pathway↓	[57]
	AS-IV	SW962	255 μM; 24 h	Bax, and cleaved caspase-3↑; Bcl-2, Bcl-xl, and TGF-β1/FAK/Akt pathway↓	[39]
	DS	A549	25, 50, 100, and 200 μM; 72 h; A549: IC_50_ 20.9 μM	PARP and caspase-3↑; EGFR and STAT3↓	[58]
	AS	DU145	20, 50, and 100 nM; 72 h	IRE1, p-PERK, AFT4, AFT6, BiP, CHOP, and caspase-12 mRNA↑	[59]
	AS-IV	A549^Cis^, H1299^Cis^	3, 5, 10, and 20 nM; 24 h	PERK, LC3 II/I ratio, GRP78, and Beclin1↓	[60]
	Lupeol	HEp2, UPCI	50 μM; 24h; Hep-2: IC50 53.51 μM, UPCI: IC50 52.44 μM	p53 and p16↑; Cyclin D1↓	[38]
	AS and CGA	4T1, EMT6, BT549, MDA-MB-231, SPF-BALB/c mice	2, 6 mM; 72 h; 4T1: IC_50_ 6.7 mM, EMT6: IC_50_ 5.6 mM, BT-549: IC_50_ 7.3 mM, MDA-MB-231: IC_50_ 1.8 mM 20 mg/g; 21 days; p.o.	RTK, VEGF, EGF, IL-10, TGF-β, and CD34↓	[61]
	AS-IV	SMMC7721, Huh7	200 μM; 48 h	IL11/STAT3 signaling↑; lncRNA-ATB↓	[62]
	Lupeol	H1299, A549, H460, WI38	125 and 250 μM; 72 h	PARP↑; EGFR and STAT3↓	[63]
	HDG	MCF7, MDA-MB-231	0.2, 0.8, 4.2, and 21.2 μM; 24 h	caspase-3 and caspase-9↑; Apaf-1 and Cyt C↓	[51]
	AS-IV	ULM1, ULM2, SD rats	1, 10, 50, 100, 200, 300, and 400 µM; 24 h; ULM1: IC_50_ 205.9 µM ULM2: IC_50_ 215.0 µM 0.5, 4 mg/kg; 10 weeks; i.p.	IDO1↑; PTEN/PI3K/Akt pathway↓	[64]
	DS	MCF7, BGC823, MGC803, Murine H22 hepatoma allograft model	0.01, 0.1, 1, 3, 10, 30, and 100 mΜ; 48 h; MGC803: IC_50_ 19.96 μM BGC823: IC_50_ 3.13 μM, MCF-7: IC_50_ 16.95 μM 0.5, 2.5 mg/kg; 7 days; p.o.	ROS production, and LC3-II↑	[45]
	Lupeol	MNNG/HOS, MG63	30 mM; 24 h	miR-212-3p↑; HMGA2↓	[65]
	HDG	SNU1041, SNU1066, SNU1076, BALB/c nu/nu mice	50 and 100 μM; 72 h; 200 mg/kg; 35 days; i.p.	cleaved PARP, cleaved caspase-3, and Bax↑; Nrf2-ARE pathway↓	[66]
Inhibition of cell migration and invasion	AS-IV	U251	25, 50, and 100 μM; 48 h	vimentin, N-cadherin, β-catenin, Cyclin D1, and Wnt/β-catenin pathway↓	[67]
	AS-IV	Huh7, MHCC97-H	1, 6, 12, 62, and 125 μM; 24, 48, and 72 h	E-cadherin↑; vimentin, α-SMA, N-cadherin, and Akt/GSK-3β/β-catenin pathway↓	[68]
	AS-IV	HCC827, A549, NCI-H1299	4, 8, 15, and 30 nM; 48 h	cleavage of caspase-3 and Bcl-2↑; Bax and Akt/GSK-3β/β-catenin pathway↓	[50]
	Lupeol	U2OS	0, 5, 10, 15, 20, and 25 μM; 12 or 24 h	uPA, MMP-2, MMP-9, N-cadherin, β-catenin, p38, and PI3K/Akt/GSK-3β pathway↓	[69]
	AS-IV	A549	5, 10, and 20 μM; 24 h	MMP-2, MMP-9, integrin β1, and PKC-α-ERK1/2-NF-κB pathway↓	[70]
	AS-IV	SiHa, BALB/c nude mice	62, 255, and 1000 μM; 24 h; SiHa: IC_50_ 800.39 μM 120 mg/kg; 21 days; i.g.	TGF-β1, N-cadherin, Vimentin, and E-cadherin↑; MAPK and PI3K↓	[71]
	AS-IV	A549	5, 10, and 20 μM; 24 h	MMP-2, MMP-9, integrin β1, and PKC-α-ERK1/2-NF-κB pathway↓	[70]
	AS-IV	SW962	125, 255 μM; 24 h	E-cadherin, TGF-β1, FAK, and Akt↓	[72]
	AS-IV	Immortalized mouse podocyte cell line, C57BL/6 J mice	25, 50, and 100 μM; 48 h; 40 mg/kg; 12 weeks; i.g.	Nephrin, E-cadherin, and SIRT1↑; NF-κB, TGF-β, N-cadherin, α-SMA, Beclin 1, and LC3 II↓	[73]
	AS-IV	HGC27, MKN45, nude mice	10, 20, and 40 μM; 24, 48 h; 40 mg/kg; 21 days; i.p.	EIF4A1↑ Regulate circDLST/miR489-3p/EIF4A1 axis. circDLST↓	[74]
	AS-IV	SGC7901, MGC803	6, 12, 31, and 62 μM; 24 h	miR-195-5p and miR-424-5p↑; PD-L1↓	[75]
	AS-IV	SW480	6, 12, 31, and 62 μM; 24 or 48 h	miR-134↑; CREB1↓	[76]
	AS-IV	MCF7, MDA-MB-231, MDA-MB-468, BALB/c nude mice	25, 50, and 100 μM; 48 h; 20 mg/kg; every three days, 14 days; i.p.	TRHDE-AS1↑; MMP-2 and MMP-9↓	[77]
	AS-IV	SMMC7721, Huh7	200 μM; 48 h	lncRNA-ATB↓	[62]
	SsaI	B16F10, NIH/3T3, C57BL/6J male mice	25, 50, and 75 μM; 24 h	α2, 3-linked sialic acids↓	[78]
	SsaI	MCF7, MDA-MB-231	5, 25, 50, and 100 μM; 48, 72 h;	α2,3-sialylations and ST3Gal IV↓	[79]
	AS-IV	MDA-MB-231, BALB/c nude mice	12, 25, 38, and 50 μM; 24 h; 20 mg/kg; every three days, 6 weeks; i.p.	p-ERK1/2, p-JNK, and Vav3↓, Rac1 signaling↓	[80]
Regulation of autophagy	DS	A549, NCI–H460, NCI–H23, L132, female Wistar	25, 50, 100, and 200 μM; 24 h; 2.5, 10 mg/kg; 28 days; i.g.	MDA, CA 15-3, TC, TG, and HDL-C↓	[81]
	Isoalantolactone	MDA-MB-231	1, 2, and 4 µM; 24 h	MMP-2, MMP-9, p38, MAPK, NF-κB, and p65↓	[82]
	AS-IV	THP1, BALB/c nude mice	5, 10, 25, 50, and 100 μM; 24 h; 40 mg/kg; 20 days; i.p.	Akt/Foxo1 and TGF-β↓	[83]
Enhance cellular immunity	AS-IV	A549, H1975, BALB/c nude mice	20 mg/kg; 20 days; p.o.	MCM5/HDAC1, HDAC1, and MCM5↓	[84]
	AS-III	bEnd.3	10, 100, and 1000 nM; 24 h	EGFR, ERK1/2, p38, and AKT↑ Inhibit PMA-induced EPCR shedding through MAPKs and PKC pathway.	[85]
	AS-IV	HUVECs, SD rats	10, 20, 40, 80, and 160 μM; 18 h; 20, 50 mg/kg; 2 weeks; i.g.	VEGF and PTEN/PI3K/Akt pathway↑	[86]
	AS-II	Bel7402, Bel7402/FU	0, 40, 80, 160, and 320 μM; 48 h	LC3-II, Beclin-1, and MAPK/mTOR pathway↓	[87]
	Astragalus–Scorpion	LNCap, BALB/c nude mice	A (1.17, 2.54, 3.9 g/kg); S (0.39, 0.585, 0.78 g/kg); 4 weeks; i.g.	GDPD4-2↑; PI3K, Akt, and mTOR↓	[88]
	AS-IV	SiHa, HeLA, BALB/c nude mice	5, 25, and 50 μM; 12 h; 12.5, 25, and 50 mg/kg; 35 days; i.g.	DCP1A and TMSB4X↑; LC3I/II↓	[89]
	AS-IV	A549, H1299, A549^cis^, H1299^cis^	3, 5, 10, and 20 nM; 24 h	PERK, LC3 II/I ratio, GRP78, and Beclin1↓	[60]
	AS-IV	RAW264.7	31, 62, and 125 μM; 24 h	NLRP3↑; IL-1β, IL-18, ROS, and TLR4/NF-κB pathway↓	[90]
	AS-IV	SW962	125, 255, 510, 765, and 1020 μM; 24 h	Induce G0/G1 phase arrest. TGF-βRII and Smad4↑	[39]
	AS-IV	SD rats	40, 80 mg/kg; 7 days; p.o.	caspase-1, IL-1β, and IL-18↑; NLRP3↓	[91]
	AS-IV	Kunming mice, the human lung fibroblasts cell line	6, 12, 25, 50, 100, 200, and 400 μM; 24 h; 50, 100, and 200 mg/kg; 8–35 days; i.g.	LC3B-II/LC3B-I ratio↑; Col-I, Col-II, ATG7, Ras, Raf, MEK, ERKs, CD3 T cells, and CD68 T cells↓	[92]
	DS	LNCap, PC3	5, 10, 20, 40, and 80 μM; 48 h	cleaved caspase-3, cleaved caspase-9, Bax, LC3II/LC3I ratio, Beclin 1, and JNK↑	[93]
	AS-IV	A549	1, 25, 62, and 125 nM; 24 h	CTLs↑; Tregs, IDO, and Akt/mTOR pathway↓	[94]
Combination therapy	AS-IV	A549, H1299, THP1, C57BL/6 J	80 nM; 48 h; 40 mg/kg; 21 days; i.g.	AMPK, IL-13, and IL-4↓; suppress M2 polarization of macrophages.	[95]
	AS-IV	THP1, SKOV3	1, 6, and 12 μM; 24 h	TGF-β, MMP-9, IL-10, HMGB1, and TLR4↓	[96]
	AS-IV	THP1, HeLa, SiHa	20, 40, 60, and 80 μM; 24 h; HeLa: IC_50_ 56.70 μM THP1: IC_50_ 85.34 μM	CD163, IL-10, TGFβ, CD206, p-Smad2, and p-Smad3↓	[97]
	AS-III	CT26, BALB/c mice	4, 12, 20, and 40 nM; 48 h; 50 mg/kg; 2-day intervals five times; i.v.	NKG2D, Fas, IFN-γ, and T-Bet↑	[72]
	Lupeol	N87, BGC823, HGC27	1, 2, 4, 7, 14, 29, 58, 116, 232, and 464 μM; 72 h	PFP, CD107a, IFN-γ, Wnt/β-catenin, PI3K/Akt, NK cells, NO, and TLR4/NF-κB pathway↑	[98]
	AS-IV	RAW264.7	31 to 125 μM; 24 h	p38, ERK, and JNK↓	[99]
	AS-IV	SD rats	50, 100 mg/kg; 10 weeks; p.o.	p53 and TP53↑; MCT1, MCT4, HIF-1α, and CD147↓	[100]
	AS-IV	BGC823	10, 20, and 40 μM; 72 h	SOX2, NANOG, and miR-214↑; GCAFs and miR301a↓	[101]
	AS-IV	3LL-luc, C57BL/6	40 mg/kg; 4, 8, and 12 days; p.o.	CTLs↑; Tregs, IDO, and Akt/mTOR pathway↓	[94]
	AS-IV	CT26, BALB/c female mice	10, 50, and 100 nM; 48 h; 15 mg/kg; once every three days, three times; i.p.	B7-H3, CyclinD1, and CDK4↓	[35]
	AS-IV	SiHa, HeLa	5, 25, and 50 μM; 12 h; HeLa: IC_50_ 0.49 ± 0.03 mM, SiHa: IC50 0.27 ± 0.03 mM; 12.5, 25, and 50 mg/kg; 35 days; i.g.	DCP1A and TMSB4X↑; LC3I/II↓	[89]
	AS-IV	THLE2, Huh7, SMMC7721, BALB/c nude mice	12, 25, 50, and 100 μM; 4 h; 50, 100, and 150 mg/kg; 40 days; i.g.	CNDP1↑; PD-L1 and miR-135b-5p↓	[102]
	AS-IV/β-CD IC	CTLL2, 4T1, NIH3T3, BALB/c nude mice	0.6, 1.2, 2.4, 4.8, and 9.6 μM; 24 h; 1.2, 1.6 mg/kg; 6 days; i.p.	SOD↑; MDA↓	[103]
	AS-IV	SW620, HCT116	6, 12, 25, 62, and 125 nM; 48 h	miR-29c↑; B7-H3, and NF- κB↓	[44]
	AS-IV	A549^cis^, H1299^cis^	2, 5, 10, and 20 nM; 24 h	PERK, LC3 II/I, GRP78, and Beclin1↓	[60]
	HDG	NCI-H1299, NCI-H1975, BALB/c nude mice	50 μM; 4 or 24 h; NCI-H1299: IC_50_ 101.4 Nm, NCI-H1975: IC_50_ 22.61 nM 25 mg/kg; 11 days; i.h.	LC3-II/LC3-I ratio↑; p62↓	[104]
	AS-II	Bel7402, Bel7402/FU	40, 80, 160, and 320 μM; 48 h;	LC3-II, Beclin-1, and MAPK/mTOR pathway↓	[87]
	AS-IV	MG63, 143B, BALB/c nu/nu mice	20, 40, 60, 80, and 100 μM; 48 h; 20 mg/kg; 28 days; i.g.	cleaved caspase-8, cleaved caspase-3, cleaved PARP, Fas, and FasL expression↑	[54]
	AS-IV	LNCap, PC3	10 μM; 72 h; 40 mg/kg; 24 days; i.p.	E-cadherin↑; p-IĸB, p-AKT, p-p65, N-cadherin, and Vimentin↓	[105]
	AS-IV	A549, H1975, BALB/c nude mice	20 mg/kg; once every two days, 20 days; i.g.	MCM5/HDAC1, HDAC1, and MCM5↓	[84]
	AS-IV	SW480	0, 6, 12, 31, and 62 μM; 24 or 48 h	miR-134↑; CREB1↓	[76]
	AS-IV	A549, HCC827, NCI-H1299	1, 3, 6, 12, 25, and 50 nM; 48 h	B7-H3↓; increase cisplatin cytotoxicity	[106]
	AS-IV	HCT116, SW480, NCM460	3, 6, 9, 12, and 18 nM; 48 h	NOTCH3↓	[107]
	AS-IV	NCI-H1299, HCC827, A549	3, 7, 15, and 30 nM; 48 h	Increase gefitinib sensitivity. SIRT6↑	[108]
	AS-IV	MCF7, MCF10A, MDA-MB-231	10, 20, 30, 40, 50, 60, 70, 80, and 90 μM; 48 h	eNOS/NO/ONOO^−^ pathway↑; phosphorylation of CAV-1 and ERK/JNK↓	[109]
	AS-IV	HepG2, H22, BALB/c nude mice	0.4, 4, and 40 μM; 24 h; 50 mg/kg; 14 days; p.o.	MRP2↓	[110]
	AS-IV	Bel7402, Bel7402/FU	0.1, 0.2, 0.4, and 0.8 mM; 48 h	P-gp and mdr1↓	[111]
	AS-IV	Bel7402/FU	0.1 mM; 24 h	JNK, c-Jun, and APD-1 DNA binding activity↓	[112]
	AS-IV	4T1, EMT6, BT549, MDA-MB-231, SPF- BALB/c nude mice	2.5 and 3 mM; 1.6 and 2.5 mM; 2 and 2.5 mM; 72 h; 4T1: IC50 6.7 mM, EMT6: IC50 5.6 mM, BT-549: IC50 7.3 mM, MDA-MB-231: IC50 1.8 mM 20 mg/g; 21 days; p.o.	RTK, VEGF, EGF, IL-10, TGF-β, and CD34↓	[61]
	AS-IV	NRCMs, C57BL/6	40 mg/kg; 4 weeks; i.g.	MDA, 8-OhdG, NOX2, and NOX4↓	[113]
	DS	A549, NCI–H460, NCI–H23, L132; female Wistar rats	25, 50, 100, and 200 μM; 24, 48, and 72 h; 2.5, 10 mg/kg; 28 days; i.p.	MDA, CA 15-3, TC, TG, and HDL-C↓	[81]
	AS-IV	BT549, MDA-MB-231, SPF- BALB/c nude mice	40 mg/kg; 4 weeks; i.g.	Nrf2, ARE-luciferin, BCRP, ATP, and GPx4↑	[114]
	HDG	Human LoVo cancer cell	1.0, 2.0, and 4.0 μM; 48 h LoVo: IC_50_ 1.17 μM	Bax↑; Bcl-2 and Bcl-xL↓	[53]
	Lupeol	OXA-R LoVo cell	50 μM; 24 h	ER stress↑; ABCG2↓	[52]
	CAG	SNU1, SNU16	50 μM; 24 h; SNU16: IC_50_ 5 μM	caspase-3 and PARP cleavage↑; p-STAT3, STAT3, JAK1, and JAK2↓	[37]
	AS-IV	SD rats	10 mg/kg; 5 weeks; i.g.	type I/III collagens, TGF-β, NOX2, and NOX4↓; promote nuclear Nrf2 level.	[115]
	AS-IV	C57BL/6J	20 mg/kg; 5 days/week, 6 weeks; i.g.	SIRT1↑; NLRP3, caspase-1/GSDMD, and caspase-3/GSDME↓	[116]
	AS-IV	SD rats	40, 80 mg/kg; 1 week; i.g.	NLRP3 and pro-inflammatory cytokines↓	[91]
	HDG	pTEC cells, C57BL/6J	21, 42, and 63 μM; 20, 40 mg/kg; 3 days; i.p.	Axin2/β-catenin and lncRNA-A330074k22Rik↓	[117]

↓, inhibit or downregulate; ↑, activate or upregulate. IC_50_, half maximal inhibitory concentration; i.g., intragastrical administration; i.p., intraperitoneal injection; i.v., intravenous injection; p.o., oral administration; i.h., subcutaneous injection.

## Data Availability

No new data were created or analyzed in this study.
